# Empagliflozin and Dapagliflozin Outcomes in Heart Failure

**DOI:** 10.1001/jamanetworkopen.2025.46865

**Published:** 2025-12-04

**Authors:** Seonghyeon Bu, Mi-Hyang Jung, Dongjae Lee, You-Mi Hwang, Jung Sun Cho, Jeong-Eun Yi, Hwajung Kim, Seoree Kim, Dukmoon Chung, Dong-Ho Shin, Nay Aung, Hyo-Suk Ahn

**Affiliations:** 1 Division of Cardiology, Department of Internal Medicine, Uijeongbu St Mary’s Hospital, College of Medicine, The Catholic University of Korea, Seoul, Republic of Korea; 2 Catholic Research Institute for Intractable Cardiovascular Disease, College of Medicine, The Catholic University of Korea, Seoul, Republic of Korea; 3 Division of Cardiology, Department of Internal Medicine, Seoul St Mary’s Hospital, College of Medicine, The Catholic University of Korea, Seoul, Republic of Korea; 4 Division of Cardiology, Department of Internal Medicine, Incheon St Mary’s Hospital, College of Medicine, The Catholic University of Korea, Seoul, Republic of Korea; 5 Division of Cardiology, Department of Internal Medicine, St Vincent’s Hospital, College of Medicine, The Catholic University of Korea, Seoul, Republic of Korea; 6 Division of Cardiology, Department of Internal Medicine, Daejeon St Mary’s Hospital, College of Medicine, The Catholic University of Korea, Seoul, Republic of Korea; 7 Division of Cardiology, Department of Internal Medicine, Eunpyeong St Mary’s Hospital, College of Medicine, The Catholic University of Korea, Seoul, Republic of Korea; 8 Division of Cardiology, Department of Internal Medicine, Yeoido St Mary’s Hospital, College of Medicine, The Catholic University of Korea, Seoul, Republic of Korea; 9 Division of Cardiology, Department of Internal Medicine, Bucheon St Mary’s Hospital, College of Medicine, The Catholic University of Korea, Seoul, Republic of Korea; 10 Department of Internal Medicine, Graduate School, The Catholic University of Korea, Seoul, Republic of Korea; 11 Department of Internal Medicine, Yonsei University College of Medicine, Seoul, Republic of Korea; 12 William Harvey Research Institute, Barts and The London Faculty of Medicine and Dentistry, Queen Mary University of London, London, United Kingdom

## Abstract

**Importance:**

Sodium-glucose cotransporter-2 inhibitors have emerged as important therapeutic options for heart failure (HF). However, their comparative clinical effectiveness remains uncertain.

**Objective:**

To compare the outcomes associated with dapagliflozin and empagliflozin use in patients diagnosed with HF.

**Design, Setting, and Participants:**

This cohort study used a clinical data warehouse platform shared by 8 medical centers affiliated with The Catholic University of Korea to screen all patients who were diagnosed with HF between January 2021 and November 2023 at these 8 medical centers. Patients were taking either dapagliflozin or empagliflozin and underwent transthoracic echocardiography. One-to-one propensity score matching was performed to ensure comparable baseline characteristics between groups. The propensity score–matched cohort was stratified by left ventricular ejection fraction (LVEF) into subgroups: HF with reduced ejection fraction group had an LVEF of 40% or lower, HF with mildly reduced ejection fraction group had an LVEF of 41% to 49%, and HF with preserved ejection fraction group had an LVEF of 50% or higher. Statistical analyses were performed from December 2023 to July 2025.

**Exposure:**

All patients received either dapagliflozin or empagliflozin.

**Main Outcomes and Measures:**

The primary outcome was a composite of cardiovascular death or hospitalization for heart failure. Secondary outcomes included the individual primary outcome components, all-cause death, and cardiovascular hospitalization.

**Results:**

After propensity score matching, the balanced cohort included 4930 patients (2465 each in the dapagliflozin and empagliflozin group; mean [SD] age, 68.8 [13.4] years; 2944 males [59.7%]). The median (IQR) follow-up duration was 16.0 (8.0-27.0) months. In the propensity score–matched cohort, dapagliflozin and empagliflozin showed no significant difference in the primary outcome: a composite of cardiovascular death or HF hospitalization occurred in 9.8% of patients (241 of 2465) taking dapagliflozin vs 9.3% of patients (229 of 2465) taking empagliflozin (adjusted hazard ratio [AHR], 0.99; 95% CI, 0.83-1.19; *P* = .95). The results did not change after stratifying the cohort by LVEF 40% or lower (14.9% [126 of 844] vs 15.4% [132 of 855]; AHR, 1.06 [95% CI, 0.83-1.35; *P* = .64]), LVEF 41% to 49% (5.0% [17 of 343] vs 6.3% [22 of 350]; AHR, 1.28 [95% CI, 0.68-2.42; *P* = .45]), and LVEF 50% or higher (7.7% [98 of 1278] vs 6.0% [75 of 1260]; AHR, 0.80 [95% CI, 0.60-1.09; *P* = .32]), without between-group heterogeneity (*P* for interaction = .32). For the secondary outcomes, there were also no significant differences between the dapagliflozin and empagliflozin groups.

**Conclusions and Relevance:**

In this cohort study of patients with HF, dapagliflozin and empagliflozin had similar clinical outcomes in HF management. Further research and clinical trials are necessary to validate these findings and inform clinical decision-making.

## Key Points

**Question:**

Are the clinical outcomes of dapagliflozin and empagliflozin comparable among patients with heart failure (HF) across the full spectrum of ejection fraction in a multicenter clinical setting?

**Findings:**

In this cohort study of 4930 patients with HF, dapagliflozin and empagliflozin showed no significant difference in the composite outcome of cardiovascular death or HF hospitalization, regardless of left ventricular ejection fraction, over a 16-month follow-up period.

**Meaning:**

These findings suggest that dapagliflozin and empagliflozin offer comparable effectiveness in the management of HF in routine clinical practice; further research is necessary to validate these findings.

## Teaser

This cohort study compares the outcomes associated with use of dapagliflozin and empagliflozin in patients with heart failure.

## Introduction

Heart failure (HF) imposes a substantial burden on patients and health care systems worldwide. Sodium-glucose cotransporter-2 (SGLT2) inhibitors, including dapagliflozin and empagliflozin, have shown promising results in HF management. In a large clinical trial targeting patients with type 2 diabetes, SGLT2 inhibitors reduced the risk of HF hospitalization.^1,2^ Subsequently, a large clinical trial targeting patients with HF with reduced ejection fraction (HFrEF), with or without diabetes, showed that SGLT2 inhibitors reduced the risk of cardiovascular (CV) death and HF hospitalization.^3,4^ Accordingly, the 2021 European Society of Cardiology, 2022 American College of Cardiology, and 2022 Korean Society of Heart Failure guidelines recommend adding dapagliflozin or empagliflozin to the existing 3-drug therapy, formulating a new 4-drug therapy for HFrEF, regardless of diabetes status.^5,6,7^

SGLT2 inhibitors also reduced the risk of CV death and HF hospitalization in a large clinical trial targeting patients with HF with mildly reduced ejection fraction (HFmrEF) or with preserved ejection fraction (HFpEF).^8,9^ Accordingly, adding empagliflozin or dapagliflozin to drug therapy is recommended for patients with HF across all ranges of left ventricular ejection fraction (LVEF).^10^ Through these randomized clinical trials, SGLT2 inhibitors have been confirmed to induce CV effects and to have hypoglycemic mechanisms. However, in clinical trials for HFrEF management, there are discrepancies between dapagliflozin and empagliflozin regarding CV death outcomes.^3,4,11,12,13,14,15,16^

To date, few comparative studies regarding the CV outcomes of dapagliflozin and empagliflozin in patients with HF based on LVEF exist. Moreover, it is unclear whether differences exist between the 2 drugs within the same class. Therefore, there remains difficulty in drug decision-making in clinical practice. This multicenter, population-based cohort analysis conducted in South Korea aimed to compare the outcomes associated with dapagliflozin and empagliflozin use in patients diagnosed with HF.

## Methods

The Catholic University of Korea Institutional Review Board approved this cohort study and waived the informed consent requirement because the study was conducted retrospectively using anonymously coded data. The study complied with the Declaration of Helsinki.^17^ We followed the Strengthening the Reporting of Observational Studies in Epidemiology (STROBE) reporting guideline.

### Data Source

This cohort study analyzed data from a clinical data warehouse (CDW) platform encompassing 8 medical centers affiliated with The Catholic University of Korea. These 8 hospitals share a standardized electronic medical record (EMR) system, which enables the integration of clinical information into the CDW. The CDW was established as a web-based platform and provides anonymized patient datasets, including visit records, diagnostic codes, prescriptions, laboratory results, imaging studies, functional tests, and clinical forms. The platform allows researchers to define study cohorts based on researcher-specified eligibility criteria and to retrieve patients who meet these criteria. Data retrieval is permitted after obtaining approval from both the institutional review board and the institutional data governance committee. The South Korean health care system operates under a single-payer, mandatory National Health Insurance Service that covers all residents.

### Study Population

Using the *International Statistical Classification of Diseases and Related Health Problems, Tenth Revision (ICD-10)* codes, we screened all patients who were diagnosed with HF from January 2021 to November 2023, prescribed either empagliflozin or dapagliflozin, and underwent transthoracic echocardiography across all departments of the 8 tertiary hospitals affiliated with The Catholic University of Korea. Data were collected directly from the EMR system via the CDW. Patients could have been treated in the cardiology department, specialized HF clinics, or other relevant departments. This time frame aligned with the January 2021 approval by the Ministry of Food and Drug Safety of Korea of the use of SGLT2 inhibitors (dapagliflozin and empagliflozin) for HF.

We included patients aged 19 years or older who were prescribed either dapagliflozin or empagliflozin. Overall, 6964 patient records were analyzed. Treatment was defined as at least 1 prescription of dapagliflozin or empagliflozin documented in the EMR. The date of the first prescription was considered the index date, and patients were followed up thereafter.

### Study Design and Outcomes

The study population was categorized into 2 groups based on the prescribed medication—dapagliflozin or empagliflozin—and followed up throughout the study period. The population was further stratified into 3 groups based on the baseline LVEF, as follows: patients with LVEF of 40% or lower formed the HFrEF group, with LVEF of 41% to 49% formed the HFmrEF group, and with LVEF of 50% or higher formed the HFpEF group. The baseline demographic data included age, sex, hypertension status, diabetes status, creatinine level, N-terminal pro–brain natriuretic peptide (NT-proBNP) level, LVEF assessed using transthoracic echocardiography, body weight, and concomitant prescribed medications. Diabetes referred to type 2 diabetes, excluding type 1 diabetes. The definitions of the diagnoses are provided in eTable 1 in Supplement 1.

The primary outcome was a composite of CV death or HF hospitalization, analyzed as the first event occurring after starting each medication. The secondary outcomes included each component of the primary outcome, all-cause mortality, and hospitalization for CV events (defined in eTable 2 in Supplement 1). As an exploratory analysis, safety outcomes including any adverse events (urinary tract infection, fracture, amputation, diabetic ketoacidosis, and hypoglycemia) were also assessed using data from the EMR and compared between groups. Adverse events were defined as newly coded corresponding *ICD-10* codes after the medication prescription.

### Statistical Analysis

Baseline characteristics were expressed as means (SDs), medians (IQRs), or numbers (percentages). Continuous variables were compared using independent *t* tests or the Wilcoxon rank-sum test depending on data normality, while categorical data were analyzed using the χ^2^ test. The baseline creatinine level, NT-proBNP level, LVEF, and body weight were collected as the earliest results within the study period. Follow-up data were recorded as the latest results before the study’s end date, and the intervals between these data varied. To minimize differences between the analysis cohorts, a 1-to-1 propensity score matching (PSM) analysis was conducted to compare the dapagliflozin and empagliflozin groups. The propensity score was estimated using logistic regression, with all baseline variables included in the model. The covariates included age; sex; hypertension status; diabetes status; baseline creatinine level, NT-proBNP level, LVEF, and body weight; and all concomitant medications. We used a greedy-matching algorithm within a caliper width equal to 0.1 of the SD of the logit of the PSM. Missing data in the overall cohort were less than 4%, and no missing data were observed after PSM. Covariate balance between the medication groups was assessed using standardized mean differences, with values less than 0.1 considered indicative of adequate balance. We also compared the baseline characteristics of matched and unmatched patients.

The association between treatment medications and outcomes was evaluated using a Cox proportional hazards regression model. Cumulative event rates were estimated using Kaplan-Meier curves and compared using the log-rank test. We conducted survival analysis that treated the exposure as a time-dependent covariate. A paired *t* test evaluated within-group changes in continuous variables, including serum creatinine levels, NT-proBNP levels, LVEF, and body weight.

For sensitivity analysis, we additionally performed an analysis using inverse probability of treatment weighting on the full study population. Subgroup analyses were conducted in the cohort who underwent PSM using a Cox proportional hazards regression model. As an exploratory analysis, we also evaluated outcomes in patients with HF with improved ejection fraction (HFimpEF), defined as those with baseline LVEF of 40% or lower who subsequently improved in LVEF during follow-up.

All statistical analyses were performed from December 2023 to July 2025 using SPSS, version 29.0 (IBM Corp), and R, 4.2.2 (R Project for Statistical Computing). A 2-sided *P* < .05 indicated statistical significance.

## Results

eFigure 1 in Supplement 1 shows the study flowchart. Before PSM, the study cohort comprised 6964 patients with overall and LVEF-stratified baseline characteristics presented in eTables 3 and 4 in Supplement 1. After PSM, 4930 patients were included in the analysis (2465 patients each in the dapagliflozin and empagliflozin groups; mean [SD] age, 68.8 [13.4] years; 2944 males [59.7%] and 1986 females [40.3%]). The mean (SD) duration of treatment was 12.13 (10.41) months, and no patients switched between the 2 medication groups. The baseline characteristics were well balanced, as shown in Table 1. Baseline characteristics of unmatched and matched patients are presented in eTable 7 in Supplement 1. The unmatched patients showed different baseline characteristics compared with the matched patients.

**Table 1.** Baseline Characteristics of Patients After Propensity Score Matching

Characteristic	Patients, No. (%)		*P* value	SMD
	Dapagliflozin group (n = 2465)	Empagliflozin group (n = 2465)		
Age, mean (SD), y	68.7 (13.4)	68.9 (13.4)	.67	.01
Sex				
Male	1476 (59.9)	1468 (59.6)	.82	.01
Female	989 (40.1)	997 (40.4)		
Hypertension	737 (29.9)	739 (30.0)	.95	.002
Diabetes	1002 (40.6)	1003 (40.7)	.98	.001
Atrial fibrillation	663 (26.9)	649 (26.3)	.65	.01
Kidney function				
Baseline creatinine, mean (SD), mg/dL	1.11 (0.74)	1.09 (0.65)	.55	.02
Baseline impaired kidney function^a^	619 (25.1)	610 (24.7)	.77	.01
NT-proBNP				
Baseline, mean (SD), pg/mL	3120.21 (6065.49)	3105.19 (6425.66)	.93	.002
LVEF				
Baseline, mean (SD), %	47.71 (15.01)	47.91 (15.06)	.64	.01
Baseline ≤40	844 (34.2)	855 (34.7)	.65	.008
Body weight				
Baseline, mean (SD), kg	67.99 (15.08)	67.80 (14.95)	.65	.01
Medications				
β-Blocker	2014 (81.7)	2010 (81.5)	.88	.004
ARNI/ACEI/ARB	2129 (86.4)	2118 (85.9)	.65	.01
ARNI	877 (35.6)	859 (34.8)	.59	.02
ACEI	143 (5.8)	151 (6.1)	.63	.01
ARB	1578 (64.0)	1563 (63.4)	.66	.01
MRA	1352 (54.8)	1338 (54.3)	.69	.01
Digoxin	263 (10.7)	260 (10.5)	.89	.004
Vasodilators	745 (30.2)	735 (29.8)	.76	.01
Ivabradine	172 (7.0)	167 (6.8)	.78	.01
GLP-1 RA	53 (2.2)	69 (2.8)	.14	.04

Abbreviations: ACEI, angiotensin-converting enzyme inhibitor; ARB, angiotensin receptor blocker; ARNI, angiotensin receptor/neprilysin inhibitor; GLP-1 RA, glucagon-like peptide-1 receptor agonist; LVEF, left ventricular ejection fraction; MRA, mineralocorticoid receptor antagonist; NT-proBNP, N-terminal pro–brain natriuretic peptide; SMD, standardized mean difference.

SI conversion factor: To convert creatinine to micromoles per liter, multiply by 88.4; NT-proBNP to nanograms per liter, multiply by 1.

^a^ Impaired kidney function means creatinine level greater than 1.2 mg/dL.

The baseline characteristics of patients after PSM stratified according to LVEF are shown in Table 2. The HFrEF group included 1699 patients (844 in the dapagliflozin group: mean [SD] age, 66.4 [14.6] years, 569 males [67.4%]; 855 in the empagliflozin group: mean [SD] age, 67.1 [13.8] years, 589 males [68.9%]), with no significant differences between the treatment groups. The HFmrEF group included 693 patients (343 in the dapagliflozin group: mean [SD] age, 68.5 [13.1] years, 217 males [63.3%]; 350 in the empagliflozin group: mean [SD] age, 67.3 [13.7] years, 237 males [67.7%]), with no significant differences between the treatment groups. The HFpEF group included 2538 patients (1278 in the dapagliflozin group: mean [SD] age, 70.2 [12.5] years, 690 males [54.0%]; 1260 in the empagliflozin group: mean [SD] age, 70.5 [12.9] years, 642 males [51.0%]), with no significant differences between the treatment groups. The use of concomitant guideline-directed medical therapy was not significantly different between treatment groups (Table 2). Older age and female sex were mainly observed in the HFpEF group, whereas the use of β-blockers; angiotensin receptor/neprilysin inhibitor, angiotensin-converting enzyme inhibitor, and angiotensin receptor blocker; and mineralocorticoid receptor antagonist was markedly higher among patients with HFrEF compared with those with HFmrEF or HFpEF, reflecting current guideline-directed therapy. Follow-up characteristics of creatinine, NT-porBNP, LVEF, and body weight were separately presented in eTable 5 and eTable 6 in Supplement 1. Serum creatinine levels and LVEF significantly increased from baseline to follow-up, with no significant between-group difference; body weight significantly decreased from baseline to follow-up, with no significant between-group difference; and NT-proBNP levels did not significantly change from baseline to follow-up, with no significant between-group difference.

**Table 2.** Baseline Characteristics of Matched Patients Based on Left Ventricular Ejection Fraction by Medication Group

Characteristic	Patients, No. (%)									*P* value for difference
	With HFrEF (LVEF ≤40%) (n = 1699)			With HFmrEF (LVEF 41%-49%) (n = 693)			With HFpEF (LVEF ≥50%) (n = 2538)			
	Dapagliflozin (n = 844)	Empagliflozin (n = 855)	*P* value	Dapagliflozin (n = 343)	Empagliflozin (n = 350)	*P* value	Dapagliflozin (n = 1278)	Empagliflozin (n = 1260)	*P* value	
Age, mean (SD), y	66.4 (14.6)	67.1 (13.8)	.33	68.5 (13.1)	67.3 (13.7)	.23	70.2 (12.5)	70.5 (12.9)	.63	<.001
Sex										
Male	569 (67.4)	589 (68.9)	.52	217 (63.3)	237 (67.7)	.22	690 (54.0)	642 (51.0)	.13	<.001
Female	275 (32.6)	266 (31.1)		126 (36.7)	113 (32.3)		588 (46.0)	618 (49.0)		
Hypertension	158 (18.7)	166 (19.4)	.72	90 (26.2)	102 (29.1)	.39	489 (38.3)	471 (37.4)	.65	<.001
Diabetes	235 (27.8)	213 (24.9)	.17	110 (32.1)	122 (34.9)	.44	657 (51.4)	668 (53.0)	.42	<.001
Atrial fibrillation	195 (23.1)	241 (28.2)	.02	97 (28.3)	79 (22.6)	.08	371 (29.0)	329 (26.1)	.10	.28
Kidney function										
Baseline creatinine, mean (SD), mg/dL	1.16 (0.80)	1.19 (0.79)	.46	1.12 (0.80)	1.12 (0.72)	.96	1.07 (0.69)	1.02 (0.50)	.07	<.001
Baseline impaired kidney function^a^	249 (29.5)	245 (28.7)	.70	74 (21.6)	92 (26.3)	.15	296 (23.2)	273 (21.7)	.37	<.001
Baseline NT-proBNP, mean (SD), pg/mL	5548.14 (8141.26)	5533.98 (8801.68)	.97	2583.01 (4974.30)	2733.88 (5190.71)	.70	1660.96 (3808.13)	1560.22 (3788.96)	.50	<.001
Baseline LVEF, mean (SD), %	29.90 (7.42)	30.28 (6.88)	.28	45.03 (2.43)	45.17 (2.43)	.45	60.19 (5.43)	60.64 (5.89)	.05	<.001
Baseline body weight, mean (SD), kg	68.10 (16.87)	67.38 (15.24)	.36	67.43 (14.33)	69.03 (15.91)	.16	68.07 (13.99)	67.73 (14.47)	.55	.76
Medications										
β-Blocker	750 (88.9)	783 (91.6)	.06	292 (85.1)	310 (88.6)	.18	972 (76.1)	917 (72.8)	.06	<.001
ARNI/ACEI/ARB	808 (95.7)	810 (94.7)	.34	308 (89.8)	304 (86.9)	.23	1013 (79.3)	1004 (79.7)	.79	<.001
ARNI	640 (75.8)	676 (79.1)	.11	126 (36.7)	101 (28.9)	.03	111 (8.7)	82 (6.5)	.04	<.001
ACEI	67 (7.9)	78 (9.1)	.38	26 (7.6)	26 (7.4)	.94	50 (3.9)	47 (3.7)	.81	<.001
ARB	403 (47.7)	392 (45.8)	.43	234 (68.2)	243 (69.4)	.73	941 (73.6)	928 (73.7)	.99	<.001
MRA	654 (77.5)	671 (78.5)	.62	196 (57.1)	188 (53.7)	.36	502 (39.3)	479 (38.0)	.51	<.001
Digoxin	117 (13.9)	138 (16.1)	.19	31 (9.0)	21 (6.0)	.13	115 (9.0)	101 (8.0)	.38	<.001
Vasodilators	258 (30.6)	235 (27.5)	.16	99 (28.9)	120 (34.3)	.13	388 (30.4)	380 (30.2)	.91	.43
Ivabradine	132 (15.6)	124 (14.5)	.51	10 (2.9)	16 (4.6)	.25	30 (2.3)	27 (2.1)	.73	<.001
GLP-1 RA	20 (2.4)	22 (2.6)	.79	8 (2.3)	7 (2.0)	.76	25 (2.0)	40 (3.2)	.05	.84

Abbreviations: ACEI, angiotensin-converting enzyme inhibitor; ARB, angiotensin receptor blocker; ARNI, angiotensin receptor/neprilysin inhibitor; GLP-1 RA, glucagon-like peptide-1 receptor agonist; HFmrEF, heart failure and mildly reduced ejection fraction; HFpEF, heart failure with preserved ejection fraction; HFrEF, heart failure with reduced ejection fraction; LVEF, left ventricular ejection fraction; MRA, mineralocorticoid receptor antagonist; NT-proBNP, N-terminal pro–brain natriuretic peptide.

SI conversion factor: To convert creatinine to micromoles per liter, multiply by 88.4; to convert NT-proBNP to nanograms per liter, multiply by 1.

^a^ Impaired kidney function means creatinine level greater than 1.2 mg/dL.

### Outcomes

In the cohort who underwent PSM, the median (IQR) follow-up duration was 16.0 (8.0-27.0) months. The primary outcome—a composite of CV death or HF hospitalization—occurred in 241 of 2465 patients (9.8%) in the overall dapagliflozin group and 229 of 2465 (9.3%) in the overall empagliflozin group (adjusted hazard ratio [AHR], 0.99; 95% CI, 0.83-1.19; *P* = .95) (Table 3 and Figure, A). For the secondary outcomes, CV death occurred in 70 patients (2.8%) in the dapagliflozin group and 63 patients (2.6%) in the empagliflozin group (AHR, 0.94; 95% CI, 0.67-1.32; *P* = .94); all-cause death occurred in 132 patients (5.4%) and 129 patients (5.2%), respectively (AHR, 1.02; 95% CI, 0.80-1.30; *P* = .90) (Table 3 and Figure, B and C); HF hospitalization, which occurred as the first event after starting each medication, occurred in 194 patients (7.9%) and 194 patients (7.9%), respectively (AHR, 1.05; 95% CI, 0.86-1.28; *P* = .66); and CV hospitalization occurred in 323 patients (13.1%) and 310 patients (12.6%), respectively (AHR, 0.99; 95% CI, 0.85-1.16; *P* = .94) (Table 3 and Figure, D and E). After stratifying according to LVEF, the primary outcome and secondary outcomes were also similar between the treatment groups (LVEF ≤40%: 14.9% [126 of 844] vs 15.4% [132 of 855], AHR, 1.06 [95% CI, 0.83-1.35; *P* = .64]; LVEF 41%-49%: 5.0% [17 of 343] vs 6.3% [22 of 350], AHR, 1.28 [95% CI, 0.68-2.42, *P* = .45]; LVEF ≥50%: 7.7% [98 of 1278] vs 6.0% [75 of 1260], AHR, 0.80 [95% CI, 0.60-1.09; *P* = .32]), without significant between-stratified group heterogeneity (*P* for interaction = .32) (Table 4; eFigure 2 in Supplement 1). Spline analyses showed that event rates for the primary outcome increased as LVEF declined, with comparable curve shapes observed for CV death and HF hospitalization (eFigure 3 in Supplement 1).

**Table 3.** Primary, Secondary, and Exploratory Safety Outcomes for Matched Patients

Outcomes	Patients, No. (%)		Crude HR (95% CI)	*P* value	Adjusted HR (95% CI)^a^	*P* value
	Dapagliflozin group (n = 2465)	Empagliflozin group (n = 2465)				
Primary^b^	241 (9.8)	229 (9.3)	0.98 (0.82-1.18)	.87	0.99 (0.83-1.19)	.95
Secondary						
CV death	70 (2.8)	63 (2.6)	0.93 (0.66-1.31)	.69	0.94 (0.67-1.32)	.94
All-cause death	132 (5.4)	129 (5.2)	1.01 (0.80-1.29)	.91	1.02 (0.80-1.30)	.90
HF hospitalization	194 (7.9)	194 (7.9)	1.04 (0.85-1.27)	.73	1.05 (0.86-1.28)	.66
CV hospitalization	323 (13.1)	310 (12.6)	0.99 (0.85-1.16)	.89	0.99 (0.85-1.16)	.94
Exploratory safety						
Any AEs	143 (5.8)	142 (5.8)	NA	.95	NA	NA
UTI	51 (2.1)	67 (2.7)	NA	.14	NA	NA
Fracture	66 (2.7)	66 (2.7)	NA	>.99	NA	NA
Amputation	1 (<0.1)	0	NA	.32	NA	NA
Diabetic ketoacidosis	9 (0.4)	2 (0.2)	NA	.04	NA	NA
Hypoglycemia	23 (0.9)	18 (0.7)	NA	.43	NA	NA

Abbreviations: AE, adverse event; CV, cardiovascular; HF, heart failure; HR, hazard ratio; NA, not applicable; UTI, urinary tract infection.

^a^ Adjusted HR was adjusted for age, sex, diabetes, and chronic kidney disease.

^b^ Primary outcome is a composite of CV death or HF hospitalization.

**Figure.**
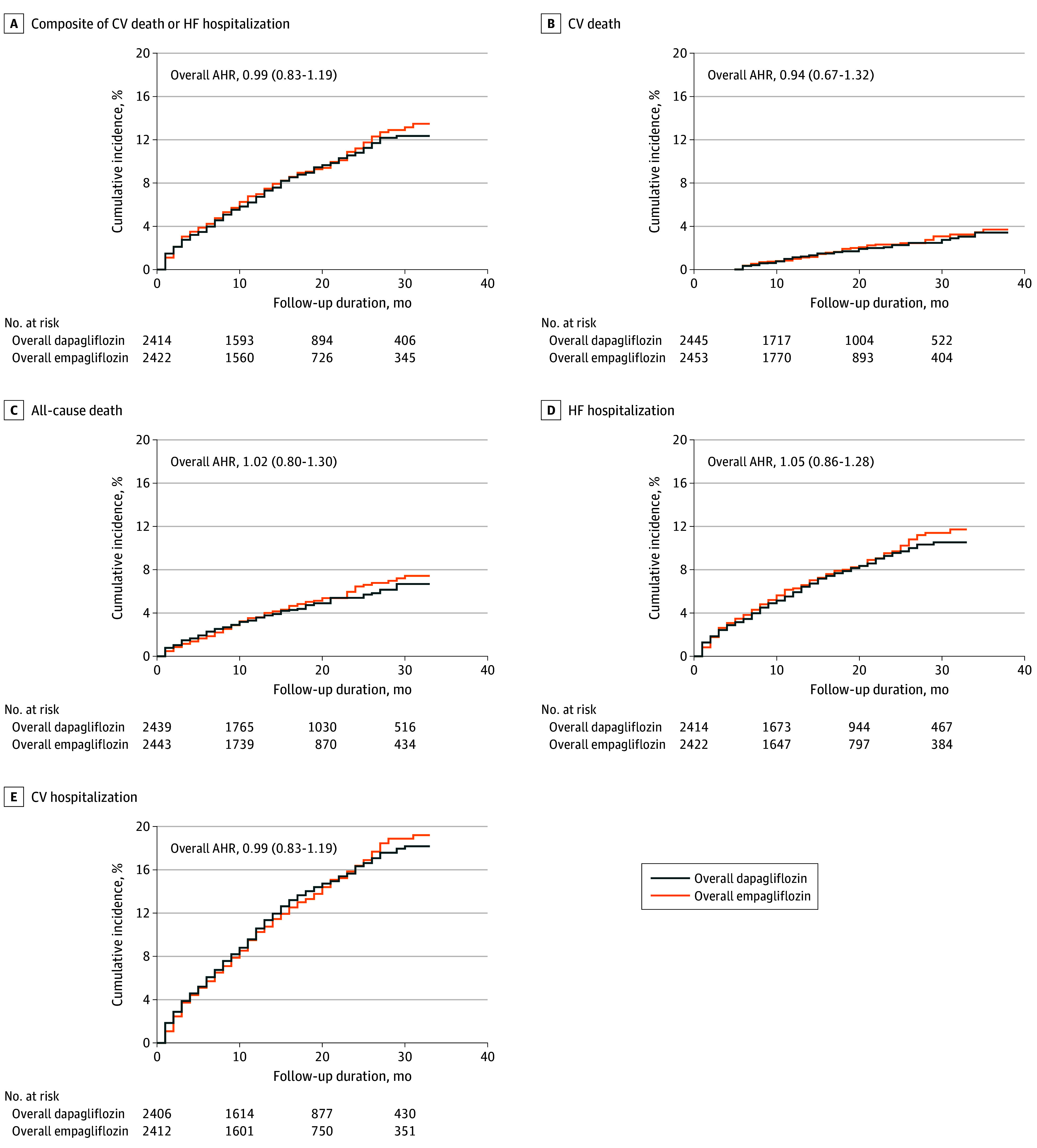
Cumulative Events Rate in the 2 Medication Groups After Propensity Score Matching AHR indicates adjusted hazard ratio; CV, cardiovascular; HF, heart failure.

**Table 4.** Primary, Secondary, and Exploratory Safety Outcomes for Matched Patients Based on Left Ventricular Ejection Fraction

Outcomes	Patients, No. (%)		Crude HR (95% CI)	*P* value	Adjusted HR (95% CI)^a^	*P* value	*P* value for interaction^b^
	Dapagliflozin group (n = 844)	Empagliflozin group (n = 855)					
**HFrEF (LVEF **≤**40%)**							
No.	844	855	NA	NA	NA	NA	NA
Primary^c^	126 (14.9)	132 (15.4)	1.05 (0.82-1.34)	.72	1.06 (0.83-1.35)	.64	.32
Secondary							
CV death	40 (4.7)	32 (3.7)	0.80 (0.50-1.27)	.34	0.83 (0.52-1.31)	.42	.43
All-cause death	56 (6.6)	48 (5.6)	0.85 (0.58-1.25)	.41	0.86 (0.59-1.27)	.86	.16
HF hospitalization	100 (11.8)	114 (13.3)	1.14 (0.87-1.49)	.35	1.15 (0.88-1.51)	.31	.20
CV hospitalization	142 (16.8)	150 (17.5)	1.04 (0.83-1.31)	.72	1.05 (0.83-1.32)	.68	.44
Exploratory safety							
Any AEs	25 (3.0)	43 (5.0)	NA	.30	NA	NA	.05
**HFmrEF (LVEF 41%-49%)**							
No.	343	350	NA	NA	NA	NA	NA
Primary^a^	17 (5.0)	22 (6.3)	1.30 (0.69-2.45)	.41	1.28 (0.68-2.42)	.45	.32
Secondary							
CV death	6 (1.7)	9 (2.6)	1.48 (0.53-4.15)	.46	1.40 (0.49-3.96)	.53	.43
All-cause death	11 (3.2)	21 (6.0)	1.95 (0.94-4.05)	.07	1.98 (0.95-4.13)	.07	.16
HF hospitalization	12 (3.5)	16 (4.6)	1.35 (0.63-2.86)	.43	1.33 (0.62-2.82)	.46	.20
CV hospitalization	40 (11.7)	43 (12.3)	1.09 (0.71-1.68)	.70	1.10 (0.72-1.70)	.66	.44
Exploratory safety							
Any AEs	18 (5.2)	19 (5.4)	NA	.92	NA	NA	.05
**HFpEF (LVEF ≥50%)**							
No.	1278	1260	NA	NA	NA	NA	NA
Primary^a^	98 (7.7)	75 (6.0)	0.82 (0.60-1.10)	.18	0.80 (0.60-1.09)	.16	.32
Secondary							
CV death	24 (1.9)	22 (1.7)	0.99 (0.55-1.76)	.96	0.98 (0.55-1.75)	.94	.43
All-cause death	65 (5.1)	60 (4.8)	0.99 (0.70-1.41)	.97	0.99 (0.70-1.41)	.95	.16
HF hospitalization	82 (6.4)	64 (5.1)	0.83 (0.60-1.15)	.27	0.82 (0.59-1.13)	.22	.20
CV hospitalization	141 (11.0)	117 (9.3)	0.99 (0.55-1.76)	.96	0.88 (0.69-1.12)	.30	.44
Exploratory safety							
Any AEs	100 (7.8)	80 (6.3)	NA	.15	NA	NA	.05

Abbreviations: AE, adverse event; CV, cardiovascular; HF, heart failure; HFmrEF, heart failure and mildly reduced ejection fraction; HFpEF, heart failure with preserved ejection fraction; HFrEF, heart failure with reduced ejection fraction; HR, hazard ratio; LVEF, left ventricular ejection fraction; NA, not applicable.

^a^ Adjusted HR is adjusted for age, sex, diabetes, and chronic kidney disease.

^b^ *P* values for interaction represent the significance of differences in treatment effect among LVEF subgroups (HFrEF, HFmrEF, and HFpEF).

^c^ Primary outcome is a composite of CV death or HF hospitalization.

As an exploratory analysis, we analyzed for another HF phenotype: HFimpEF. Patients with baseline LVEF of 40% or lower who showed improvement in LVEF were considered to represent HFimpEF, and the results are presented in eTable 8 and eTable 9 in Supplement 1. Compared with the patients in the HFrEF group, the patients of the HFimpEF group were younger age and had fewer cases of hypertension, diabetes, and impaired kidney function, indicating younger age and fewer comorbidities. The outcomes were similar between the medication groups.

In the sensitivity analysis using inverse probability of treatment weighting, the results were consistent with those of the PSM analysis, showing no significant differences between dapagliflozin and empagliflozin in the primary or secondary outcomes (eTable 10 and eTable 11, and eFigure 4 in Supplement 1).

## Discussion

This study showed clinical data on the comparative effectiveness of dapagliflozin and empagliflozin in patients with HF. The findings indicated that both medications have similar outcomes regarding CV death, all-cause mortality, HF hospitalization, and CV hospitalization across all ranges of LVEF in patients with HF.

The EMPEROR-Reduced and DAPA-HF trials showed that empagliflozin and dapagliflozin reduced the risk of CV death or HF hospitalization in patients with HFrEF.^3,4^ Subsequently, the EMPEROR-Preserved and DELIVER trials showed similar findings in patients with HFmrEF or HFpEF.^8,9^ The overall results were comparable across these trials; however, CV mortality was significantly reduced in the DAPA-HF trial but not in the EMPEROR-Reduced trial as a secondary outcome. These 2 trials were different in their inclusion criteria regarding NT-proBNP levels and LVEF.^18^ The differences in inclusion criteria might contribute to the discrepant CV outcomes. Although the DAPA-HF trial suggested a reduction in CV death, this outcome was assessed as a secondary end point in both studies and should be regarded as hypothesis-generating rather than confirmatory. The EMPEROR-Preserved and DELIVER trials included patients with similar baseline characteristics, and the clinical outcomes did not differ between the trials. In the present cohort study, there were no significant differences in the baseline characteristics including NT-proBNP level and LVEF between the dapagliflozin and empagliflozin groups across all LVEF ranges, which might explain no significant differences in the clinical outcomes. While we reported NT-proBNP level as a mean (SD) for consistency with other continuous variables and statistical analyses conducted using continuous values—an approach that we believe better reflects a clinical cohort—the value in our study was comparable to that in randomized clinical trials when expressed as a median.

Few studies have compared the outcomes of dapagliflozin and empagliflozin, and their findings lack consistency. A nationwide cohort study in South Korea demonstrated that patients with type 2 diabetes treated with dapagliflozin experienced a 12% to 17% reduced risk of incident AF compared with empagliflozin use.^14^ Conversely, a large cohort study reported that empagliflozin was associated with a 10% lower risk of the composite outcome of all-cause mortality or hospitalization compared with dapagliflozin.^12^ However, these studies had limitations, including missing data on the NT-proBNP level and LVEF. Additionally, a nationwide cohort study using Danish health care data indicated that patients with type 2 diabetes had comparable long-term kidney outcomes when treated with either dapagliflozin or empagliflozin. Nevertheless, this Danish study also limited information on the NT-proBNP level and LVEF.^19^ The present study contributes to the literature as it comprehensively analyzed clinical data from patients using dapagliflozin or empagliflozin, detailing baseline characteristics and changes in clinical parameters, such as creatinine levels, NT-proBNP levels, LVEF, and body weight. By classifying patients based on LVEF into HFrEF, HFmrEF, and HFpEF subgroups, the present study showed comparable outcomes of the 2 drugs across these subgroups. Moreover, patients with HFimpEF compared with HFrEF, were characterized by younger age and fewer comorbidities. These findings also provide evidence of the clinical characteristics of patients with HFimpEF.

### Limitations

This study had some limitations. First, although comorbidities were investigated using *ICD-10* codes, there may have been discrepancies between the true prevalence of comorbidities and cases recorded in the dataset. Second, as this study had an observational design, there may have been residual confounders. For example, we lacked data on comorbidities, such as myocardial infarction or atrial fibrillation. Third, this study primarily included Korean participants; therefore, caution is needed when generalizing these results to other ethnic groups. Finally, adherence information regarding the drugs was not included.

## Conclusions

In this cohort study of patients with HF, dapagliflozin and empagliflozin had similar clinical outcomes in the management of HFrEF, HFmrEF, and HFpEF in a clinical setting. Further research and clinical trials are necessary to validate these findings and inform clinical decision-making.

## Notes

Supplement 1. **eTable 1.** Definition of Baseline Disease Diagnoses **eTable 2.** Definition of Outcomes **eTable 3.** Baseline Characteristics of Overall Patients According to Medicine Group **eTable 4.** Baseline Characteristics of Overall Patients Based on LVEF According to Medicine Group **eTable 5.** Follow Up Characteristics of the Propensity Score Matched Patients **eTable 6.** Follow Up Characteristics of Propensity Score Matched Patients Based on LVEF According to Medicine Group **eTable 7.** Baseline Characteristics of Matched and Unmatched Patients After Propensity Score Matching (n = 6964) **eTable 8.** Baseline Characteristics of the Patients of HFimpEF **eTable 9.** Primary and Secondary Outcomes of the Patients of HFimpEF **eTable 10.** Baseline Characteristics of Patients by Weight (n = 6964) **eTable 11.** Primary and Secondary Outcomes of IPTW Patients **eFigure 1.** Study Flow Chart **eFigure 2.** Comparison of the Effect of Dapagliflozin and Empagliflozin on the Primary Outcome in the Subgroups of the Propensity Score-Matched Cohort **eFigure 3.** Spline Curve of Event Rates **eFigure 4.** Primary and Secondary Outcomes of KM Curve Using IPTW

Supplement 2.
Data Sharing Statement
