# Supplementary material for: Empagliflozin and Dapagliflozin Outcomes in Heart Failure
Source: JAMA Netw Open. 2025 Dec 4;8(12):e2546865. doi: 10.1001/jamanetworkopen.2025.46865 (PMC12679325; doi:10.1001/jamanetworkopen.2025.46865)
Supplement: Supplement 1. — eTable 1. Definition of Baseline Disease Diagnoses eTable 2. Definition of Outcomes eTable 3. Baseline Characteristics of Overall Patients According to Medicine Group eTable 4. Baseline Characteristics of Overall Patients Based on LVEF According to Medicine Group eTable 5. Follow Up Characteristics of the Propensity Score Matched Patients eTable 6. Follow Up Characteristics of Propensity Score Matched Patients Based on LVEF According to Medicine Group eTable 7. Baseline Characteristics of Matched and Unmatched Patients After Propensity Score Matching (n = 6964) eTable 8. Baseline Characteristics of the Patients of HFimpEF eTable 9. Primary and Secondary Outcomes of the Patients of HFimpEF eTable 10. Baseline Characteristics of Patients by Weight (n = 6964) eTable 11. Primary and Secondary Outcomes of IPTW Patients eFigure 1. Study Flow Chart eFigure 2. Comparison of the Effect of Dapagliflozin and Empagliflozin on the Primary Outcome in the Subgroups of the Propensity Score-Matched Cohort eFigure 3. Spline Curve of Event Rates eFigure 4. Primary and Secondary Outcomes of KM Curve Using IPTW [file jamanetwopen-e2546865-s001.pdf]

Supplemental Online Content

Bu S, Jung MH, Lee D, et al. Empagliflozin and dapagliflozin outcomes in heart failure. *JAMA Netw Open*. 2025;8(12):e2546865. doi:10.1001/jamanetworkopen.2025.46865

eTable 1. Definition of Baseline Disease Diagnoses

eTable 2. Definition of Outcomes

eTable 3. Baseline Characteristics of Overall Patients According to Medicine Group

eTable 4. eTable 4. Baseline Characteristics of Overall Patients Based on Left Ventricular Ejection Fraction by Medicine Group,

eTable 5. Follow Up Characteristics of the Propensity Score Matched Patients

eTable 6. Follow Up Characteristics of Propensity Score Matched Patients Based on LVEF According to Medicine Group

eTable 7. Baseline Characteristics of Matched and Unmatched Patients After Propensity Score Matching (n = 6964)

eTable 8. Baseline Characteristics of the Patients of HFimpEF

eTable 9. Primary and Secondary Outcomes of the Patients of HFimpEF

eTable 10. Baseline Characteristics of Patients by Weight (n = 6964)

eTable 11. Primary and Secondary Outcomes of IPTW Patients

eFigure 1. Study Flow Chart
© 2025 Bu S et al. *JAMA Network Open*

eFigure 2. Comparison of the Effect of Dapagliflozin and Empagliflozin on the Primary Outcome in the Subgroups of the Propensity Score-Matched Cohort

eFigure 3. Spline Curve of Event Rates

eFigure 4. Primary and Secondary Outcomes of KM Curve Using IPTW

This supplemental material has been provided by the authors to give readers additional information about their work.

eTable 1. Definition of baseline disease diagnoses

Diagnosis	ICD-10-CM code for definition
Heart failure	I19, I25, I31, I42, I43, I48, I50
Hypertension	I10-I15
Diabetes	E11-E14
Atrial fibrillation	I48
Urinary tract infection	N39
Fracture	S02-S92, T02, T08-10
Amputation	S08-S98,
Diabetic ketoacidosis	E10-E14
Hypoglycemia	E10-E14, Y42, T38

© 2025 Bu S et al. *JAMA Network Open*

eTable 2. Definition of outcomes

Outcome	Definition
HF hospitalization	Admission for HF, as to the first event after using each medication during the study period<br>1) HF symptoms (i.e. dyspnea, mental change, ventricular arrhythmia) as the chief complaint<br>2) Decongestion therapy by diuretics<br>3) Additional medical or mechanical therapy
CV hospitalization	All admission for cardiovascular events including HF after using each medication during study period.

CV, cardiovascular; HF, heart failure

© 2025 Bu S et al. *JAMA Network Open*

eTable 3. Baseline characteristics of overall patients according to medicine group

	Dapagliflozin (N = 3794)	Empagliflozin (N = 3170)	*P*-value
Age	67.6 ± 13.44	68.5 ± 13.46	.004
Sex			.001
Male	2293 (63.1%)	1871 (59.0%)	
Female	1401 (36.9%)	1299 (41.0%)	
Hypertension	1187 (31.3%)	1001 (31.6%)	.79
Diabetes	1771 (46.7%)	1352 (42.6%)	.001
Atrial fibrillation	986 (26.0%)	808 (25.5%)	.64
Renal function, mg/dL			
baseline creatinine	1.10 ± 0.78	1.07 ± 0.61	.033
baseline impaired renal function, n (%)[a]	946/3787 (25.0%)	727/3158 (23.0%)	.06
f/u creatinine	1.21 ± 0.96	1.17 ± 0.87	.04
f/u impaired renal function, n (%)	1093/3787 (28.9%)	810/3158 (25.6%)	.003
Δ creatinine	0.11 ± 0.93	0.10 ± 0.74	.65
NT-proBNP, pg/mL			
baseline NT-proBNP	3376.04 ± 7634.00	3004.69 ± 6271.19	.04
f/u NT-proBNP	3373.68 ± 6271.19	3061.46 ± 7200.73	.11
Δ NT-proBNP	-2.37 ± 8204.90	56.78 ± 6330.99	.75
LVEF, %			
baseline LVEF	48.61 ± 14.90	49.01 ± 14.80	.26
baseline LVEF≤40	1185/3772 (31.4%)	995/3158 (31.5%)	.94
f/u LVEF	49.77 ± 13.93	49.99 ± 14.11	.53
f/u LVEF≤40	1029/3772 (27.3%)	857/3158 (27.1%)	.89
Δ LVEF	1.16 ± 8.34	0.97 ± 7.72	.33
BW, kg			
baseline BW	68.63 ± 14.94	68.30 ± 15.01	.39
f/u BW	66.57 ± 15.22	66.24 ± 15.08	.39
Δ BW	-2.06 ± 6.52	-2.06 ± 6.62	.98
Medications, n(%)			
BB	3074 (81.0%)	2537 (80.0%)	.30
ARNI/ACEI/ARB	3230 (85.1%)	2672 (84.3%)	.33
ARNI	1207 (31.8%)	984 (31.0%)	.49
ACEI	192 (5.1%)	210 (6.6%)	.01
ARB	2510 (66.2%)	1990 (62.8%)	.003
MRA	1794 (47.3%)	1604 (50.6%)	.006
digoxin	407 (10.7%)	307 (10.3%)	.15

© 2025 Bu S et al. *JAMA Network Open*

vasodilators	1125 (29.7%)	847 (26.7%)	.007
ivabradine	250 (6.6%)	188 (5.9%)	.26
GLP1A	96 (2.5%)	87 (2.7%)	.58

[a] impaired renal function means creatinine level > 1.2 mg/dL.

BB,beta-clocker; BW, body weight; ARNI, angiotensin receptor/neprilysin inhibitor; ACEI, angiotensin converting enzyme inhibitor; ARB, angiotensin II receptor blocker; GLP1A,glucagon-like peptide-1 receptor agoni; tMRA, mineralocorticoid receptor antagonist

eTable 4. Baseline characteristics of overall patients based on left ventricular ejection fraction by medicine group,

	HFrEF (LVEF≤ 40%)			HFmrEF (LVEF 41-49%)			HFpEF (LVEF≥50%)			
	Dapagliflozin (N = 1185)	Empagliflozin (N = 995)	*P*-value	Dapagliflozin (N = 520)	Empagliflozin (N = 437)	*P*-value	Dapagliflozin (N = 2067)	Empagliflozin (N = 1726)	*P*-value	*P* difference
Age	66.3 ± 14.3	67.2 ± 13.8	.14	67.7 ± 13.15	66.7 ± 13.69	.255	68.3 ± 12.97	69.8 ± 13.07	.001	.001
Sex			.39			.56			<.001	<.001
Male	829 (70.0%)	679 (68.2%)		355 (68.3%)	306 (70.0%)		1191 (57.6%)	880 (51.0%)		
Female	356 (30.0%)	316 (31.8%)		165 (31.7%)	131 (30.0%)		876 (42.4%)	846 (49.0%)		
Hypertension	218 (18.4%)	197 (19.8%)	.41	133 (25.6%)	126 (28.8%)	.26	818 (39.6%)	671 (38.9%)	.66	.001
Diabetes	372 (31.4%)	257 (25.8%)	.004	204 (39.2%)	169 (38.7%)	.86	1179 (57.0%)	917 (53.1%)	.02	.001
Atrial fibrillation	287 (24.2%)	277 (27.8%)	.06	143 (27.5%)	102 (23.3%)	.14	553 (26.8%)	428 (24.8%)	.71	.98
Renal function, mg/dL										
baseline creatinine	1.19 ± 0.93	1.17 ± 0.76	.61	1.10 ± 0.69	1.11 ± 0.68	.82	1.05 ± 0.65	1.00 ± 0.47	.005	.001
baseline impaired renal function, n (%)[a]	361/1184 (30.5%)	278/989 (28.1%)	.23	119/518 (23.0%)	108/435 (24.8%)	.50	462/2063 (22.4%)	339/1722 (19.7%)	.04	<.001
f/u creatinine	1.27 ± 1.04	1.25 ± 0.91	.56	1.23 ± 1.00	1.20 ± 0.99	.61	1.17 ± 0.90	1.11 ± 0.81	.03	.001
f/u impaired renal function, n (%)	385/1184 (32.5%)	295/989 (29.8%)	.18	160/518 (30.9%)	110/435 (25.3%)	.06	546/2063 (26.5%)	402/1722 (23.3%)	.03	<.001
Δ creatinine	0.08 ± 0.88	0.07 ± 0.75	.87	0.14 ± 1.02	0.09 ± 0.81	.47	0.12 ± 0.88	0.11 ± 0.71	.769	.192
NT-proBNP, pg/mL										
baseline NT-proBNP	5856.07 ± 9735.96	5397.75 ± 8645.66	.26	2866.58 ± 5647.49	2696.30 ± 5178.26	.65	1779.31 ± 4388.88	1511.89 ± 3649.22	.06	.001
f/u NT-proBNP	5077.08 ± 9890.57	4598.38 ± 8920.02	.25	3127.75 ± 7344.32	2485.59 ± 5772.84	.17	2310.47 ± 6599.50	2206.60 ± 6021.01	.65	.001
Δ NT-proBNP	-778.99 ± 10360.36	-799.37 ± 7989.50	.96	261.17 ± 6487.86	-210.72 ± 5554.91	.26	531.16 ± 5864.65	694.71 ± 5107.82	.41	<.001
LVEF, %										
baseline LVEF	29.89 ± 7.46	30.43 ± 6.92	.08	45.10 ± 2.42	45.25 ± 2.40	.37	60.25 ± 5.44	60.68 ± 5.72	.02	.001
f/u LVEF	35.28 ± 10.87	34.94 ± 10.89	.47	45.97 ± 7.47	45.90 ± 6.86	.87	59.04 ± 7.90	59.70 ± 7.42	.009	.001

© 2025 Bu S et al. *JAMA Network Open*

Δ LVEF	5.38 ± 10.17	4.51 ± 10.05	.02	0.87 ± 7.12	0.65 ± 6.50	.63	-1.21 ± 6.14	-0.99 ± 5.40	.23	<.001
BW, kg										
baseline BW	68.09 ± 16.31	67.64 ± 15.38	.52	68.39 ± 14.51	69.23 ± 15.56	.41	68.92 ± 14.06	68.39 ± 14.53	.28	.13
f/u BW	65.39 ± 16.49	65.21 ± 14.84	.80	66.33 ± 14.71	66.41 ± 15.21	.93	67.26 ± 14.39	66.74 ± 15.05	.30	.001
Δ BW	2.70 ± 6.85	2.43 ± 6.81	.37	-2.07 ± 5.86	-2.82 ± 6.48	.07	-1.66 ± 6.44	-1.65 ± 6.53	.98	<.001
Medications, n (%)										
BB	1060 (89.5%)	900 (90.5%)	.44	447 (86.0%)	383 (87.6%)	.45	1555 (75.2%)	1249 (72.4%)	.05	.001
ARNI/ACEI/ARB	1130 (95.4%)	940 (94.5%)	.35	461 (88.7%)	377 (86.3%)	.27	1621 (78.4%)	1347 (78.0%)	.78	.001
ARNI	887 (74.9%)	770 (77.4%)	.17	173 (33.3%)	118 (27.0%)	.04	145 (7.0%)	96 (5.6%)	.07	<.001
ACEI	83 (7.0%)	94 (9.4%)	.04	32 (6.2%)	33 (7.6%)	.39	74 (3.6%)	83 (4.8%)	.06	<.001
ARB	615 (51.9%)	451 (45.3%)	.002	362 (69.6%)	296 (67.7%)	.53	1518 (73.4%)	1235 (71.6%)	.20	<.001
MRA	873 (73.7%)	776 (78.0%)	.02	261 (50.2%)	220 (50.3%)	.96	657 (31.8%)	606 (35.1%)	.03	.001
digoxin	176 (14.9%)	151 (15.2%)	.83	52 (10.0%)	28 (6.4%)	.05	179 (8.7%)	128 (7.4%)	.16	.001
vasodilators	369 (31.1%)	259 (26.0%)	.009	151 (29.0%)	133 (30.4%)	.64	603 (29.2%)	455 (26.4%)	.06	.001
ivabradine	192 (16.2%)	138 (13.9%)	.13	19 (3.7%)	19 (4.3%)	.58	39 (1.9%)	31 (1.8%)	.84	.001
GLP1A	25 (2.1%)	25 (2.5%)	.53	14 (2.7%)	8 (1.9%)	..38	54 (2.6%)	54 (3.1%)	.34	.36

A total of 34 patients with missing LVEF data were excluded from the analysis (22 in the dapagliflozin group and 12 in the empagliflozin group).
[a] impaired renal function means creatinine level > 1.2 mg/dL.
BW, body weight; ARNI, angiotensin receptor/neprilysin inhibitor; ACEI, angiotensin converting enzyme inhibitor; ARB, angiotensin II receptor blocker; MRA, mineralocorticoid receptor antagonist

© 2025 Bu S et al. *JAMA Network Open*

eTable 5. Follow up characteristics of the propensity score matched patients.

	Dapagliflozin (N=2,465)	Empagliflozin (N=2,465)	P-value
Renal function, mg/dL			
baseline creatinine	1.11 ± 0.74	1.09 ± 0.65	0.554
baseline impaired renal function, n(%)[a]	619 (25.1%)	610 (24.7%)	0.767
f/u creatinine	1.22 ± 0.94	1.21 ± 0.94	0.738
f/u impaired renal function, n(%)	724 (29.4%)	682 (27.7%)	0.185
Δ creatinine	0.11 ± 0.92	0.12 ± 0.81	0.907
NT-proBNP, pg/mL			
baseline NT-proBNP	3120.21 ± 6065.49	3105.19 ± 6425.66	0.933
f/u NT-proBNP	3221.48 ± 7605.46	3177.23 ± 7400.89	0.836
Δ NT-proBNP	101.27 ± 7508.13	72.04 ± 6521.34	0.884
LVEF, %			
baseline LVEF	47.71 ± 15.01	47.91 ± 15.06	0.639
baseline LVEF≤40	844 (34.2%)	855 (34.7%)	0.653
f/u LVEF	48.78 ± 14.15	48.95 ± 14.41	0.672
f/u LVEF≤40	740 (30.0%)	741 (30.1%)	0.975
Δ LVEF	1.07 ± 8.81	1.04 ± 8.29	0.906
BW, kg			
baseline BW	67.99 ± 15.08	67.80 ± 14.95	0.645
f/u BW	65.74 ± 15.25	65.58 ± 14.98	0.717
Δ BW	-2.25 ± 6.51	-2.21 ± 6.78	0.829

[a]impaired renal function means creatinine level > 1.2 mg/dL.

BW,body weight

© 2025 Bu S et al. *JAMA Network Open*

eTable 6. Follow up characteristics of propensity score matched patients based on LVEF according to medicine group.

	HFrEF (LVEF≤40%; N=1,699)			HFmrEF (LVEF 41-49%; N=693)			HFpEF (LVEF≥50%; N=2,538)			
	Dapagliflozin (N=844)	Empagliflozin (N=855)	P-value	Dapagliflozin (N=343)	Empagliflozin (N=350)	P-value	Dapagliflozin (N=1,278)	Empagliflozin (N=1,260)	P-value	P difference
Renal function, mg/dL										
baseline creatinine	1.16 ± 0.80	1.19 ± 0.79	0.46	1.12 ± 0.80	1.12 ± 0.72	0.96	1.07 ± 0.69	1.02 ± 0.50	0.07	<0.001
baseline impaired renal function, n(%)[a]	249 (29.5%)	245 (28.7%)	0.70	74 (21.6%)	92 (26.3%)	0.15	296 (23.2%)	273 (21.7%)	0.37	<0.001
f/u creatinine	1.27 ± 1.05	1.27 ± 0.95	0.91	1.19 ± 0.71	1.23 ± 1.08	0.57	1.19 ± 0.92	1.16 ± 0.88	0.36	0.004
f/u impaired renal function, n(%)	263 (31.2%)	263 (30.8%)	0.86	102 (29.7%)	89 (25.4%)	0.20	359 (28.1%)	330 (26.2%)	0.28	0.022
Δ creatinine	0.11 ± 0.91	0.09 ± 0.80	0.57	0.07 ± 0.83	0.11 ± 0.89	0.52	0.13 ± 0.94	0.14 ± 0.79	0.77	0.376
NT-proBNP, pg/mL										
baseline NT-proBNP	5548.14 ± 8141.26	5533.98 ± 8801.68	0.97	2583.01 ± 4974.30	2733.88 ± 5190.71	0.70	1660.96 ± 3808.13	1560.22 ± 3788.96	0.50	<0.001
f/u NT-proBNP	4841.07 ± 9214.44	4771.63 ± 9170.48	0.88	2941.45 ± 7117.69	2558.47 ± 5870.09	0.44	2227.05 ± 6264.77	2267.18 ± 6159.44	0.87	<0.001
Δ NT-proBNP	-707.07 ± 9338.86	-762.35 ± 8169.16	0.90	358.44 ± 6892.44	-175.40 ± 5712.53	0.27	566.09 ± 6148.89	706.96 ± 5293.87	0.54	<0.001
LVEF, %										
baseline LVEF	29.90 ± 7.42	30.28 ± 6.88	0.28	45.03 ± 2.43	45.17 ± 2.43	0.45	60.19 ± 5.43	60.64 ± 5.89	0.05	<0.001
f/u LVEF	35.23 ± 10.82	34.83 ± 10.95	0.45	45.70 ± 7.89	45.95 ± 7.33	0.66	58.56 ± 8.53	59.37 ± 7.97	0.01	<0.001
Δ LVEF	5.32 ± 10.07	4.55 ± 10.18	0.12	0.67 ± 7.52	0.78 ± 6.95	0.83	-1.63 ± 6.96	-1.27 ± 6.08	0.16	<0.001
BW, kg										
baseline BW	68.10 ± 16.87	67.38 ± 15.24	0.36	67.43 ± 14.33	69.03 ± 15.91	0.16	68.07 ± 13.99	67.73 ± 14.47	0.55	0.761
f/u BW	65.25 ± 16.88	64.87 ± 14.69	0.62	64.98 ± 14.27	65.92 ± 15.54	0.41	66.26 ± 14.33	65.97 ± 15.01	0.61	0.078
Δ BW	-2.85 ± 6.79	-2.51 ± 6.91	0.30	-2.46 ± 5.97	-3.11 ± 6.49	0.16	-1.81 ± 6.42	-1.76 ± 6.73	0.87	<0.001

[a]impaired renal function means creatinine level > 1.2 mg/dL.

© 2025 Bu S et al. *JAMA Network Open*

BW,body weight

© 2025 Bu S et al. *JAMA Network Open*

eTable 7. Baseline characteristics of matched and unmatched patients after propensity score matching (n = 6964)

	Matched (n = 4930)	Unmatched (n = 2034)	P value
Age	68.78 ± 13.42	66.03 ± 13.36	<.001
Sex			<.001
Male	2944 (59.7%)	1320 (64.9%)	
Female	1986 (40.3%)	714 (35.1%)	
Hypertension	1476 (29.9%)	712 (35.0%)	<.001
Diabetes	2005 (40.7%)	1118 (55.0%)	<.001
Renal function			
baseline	1.10 ± 0.70	1.06 ± 0.73	0.052
baseline function	1229 (24.9%)	444 (22.0%)	0.011
f/u	1.21 ± 0.94	1.14 ± 0.87	0.001
f/u function	1406 (28.5%)	497 (24.7%)	0.001
delta	0.11 ± 0.86	0.07 ± 0.80	0.059
NT-proBNT			
baseline	3112.7 ± 6247.5	3625.1 ± 9840.5	0.097
f/u	3199.4 ± 7503.1	3373.6 ± 8477.8	0.528
delta	86.65 ± 7031.4	(-251.5 ± 8895.4)	0.236
LVEF			
baseline	47.81 ± 15.03	51.24 ± 14.02	<.001
baseline (≤40)	1699 (34.5%)	481 (24.1%)	<.001
f/u	1.05 ± 8.55	1.10 ± 6.61	0.801
f/u (≤40)	1481 (30.0%)	405 (20.3%)	<.001
delta	48.87 ± 14.28	52.35 ± 13.02	<.001
BW			
baseline	67.89 ± 15.01	70.55 ± 14.64	<.001
f/u	65.66 ± 15.11	69.10 ± 15.01	<.001
delta	(-2.23 ± 6.64)	(-1.45 ± 6.23)	<.001
Medications			
BB	4024 (81.6%)	1587 (78.0%)	0.001
ARNI/ACEI/ARB	4247 (86.2%)	1655 (81.4%)	<.001
ARNI	1736 (35.2%)	455 (22.4%)	<.001
ACEI	294 (6.0%)	108 (5.3%)	0.288
ARB	3141 (63.7%)	1359 (66.8%)	0.014
MRA	2690 (54.6%)	708 (34.8%)	<.001
Digoxin	523 (10.6%)	191 (9.4%)	0.128
Vasodilators	1480 (30.0%)	492 (24.2%)	<.001

© 2025 Bu S et al. *JAMA Network Open*

Ivabradine	339 (6.9%)	99 (4.9%)	0.002

[a] impaired renal function means creatinine level > 1.2 mg/dL.

BB, beta-clocker; BW, body weight; ARNI, angiotensin receptor/neprilysin inhibitor; ACEI, angiotensin converting enzyme inhibitor; ARB, angiotensin II receptor blocker; MRA, mineralocorticoid receptor antagonist

© 2025 Bu S et al. *JAMA Network Open*

eTable 8. Baseline characteristics of the patients of HFimpEF

	HFimpEF (N=777)		
	Dapagliflozin (N=408)	Empagliflozin (N=369)	P-value
Age	64.66 ± 14.88	65.41 ± 14.60	0.48
Sex			0.97
Male	277 (67.9%)	251 (68.0%)	
Female	131 (32.1%)	118 (32.0%)	
Hypertension	70 (17.2%)	66 (17.9%)	0.79
Diabetes	95 (23.3%)	95 (25.7%)	0.43
Renal function, mg/dL			
baseline creatinine	1.11 ± 0.55	1.20 ± 0.94	0.11
baseline impaired renal function, n(%)[a]	112 (27.5%)	102 (27.6%)	0.95
f/u creatinine	1.17 ± 0.71	1.25 ± 1.02	0.24
f/u impaired renal function, n(%)	115 (28.3%)	98 (26.6%)	0.61
Δ creatinine	0.06 ± 0.62	0.05 ± 0.84	0.80
NT-proBNP, pg/mL			
baseline NT-proBNP	5196.06 ± 7620.15	5221.31 ± 8090.82	0.96
f/u NT-proBNP	3958.07 ± 8964.95	3752.40 ± 8044.08	0.74
Δ NT-proBNP	-1237.99 ± 9693.34	-1468.91 ± 7564.24	0.71
LVEF, %			
baseline LVEF	28.77 ± 7.77	29.53 ± 7.26	0.16
f/u LVEF	41.34 ± 10.70	42.31 ± 11.00	0.22
Δ LVEF	12.58 ± 9.80	12.77 ± 10.19	0.79
BW, kg			
baseline BW	69.25 ± 17.99	68.26 ± 15.83	0.42
f/u BW	66.15 ± 18.01	65.26 ± 15.83	0.780
Δ BW	-3.10 ± 7.38	-2.45 ± 6.32	0.19
Medications, n(%)			
BB	370 (90.7%)	345 (93.5%)	0.150
ARNI/ACEI/ARB	398 (97.5%)	355 (96.2%)	0.28
ARNI	323 (79.2%)	303 (82.1%)	0.30
ACEI	27 (6.6%)	32 (8.7%)	0.28
ARB	223 (54.7%)	199 (53.9%)	0.84
MRA	314 (77.0%)	290 (78.6%)	0.59
digoxin	59 (14.5%)	50 (13.6%)	0.72
vasodilators	132 (32.4%)	113 (30.6%)	0.60
ivabradine	68 (16.7%)	53 (14.4%)	0.380

[a] impaired renal function means creatinine level > 1.2 mg/dL.

BB, beta blocker; BW, body weight; ARNI, angiotensin receptor/neprilysin inhibitor; ACEI, angiotensin converting enzyme inhibitor; ARB, angiotensin II receptor blocker; MRA, mineralocorticoid receptor antagonist;

© 2025 Bu S et al. *JAMA Network Open*

eTable 9. Primary and secondary outcomes of the patients of HFimpEF

	HFimpEF					
	Dapagliflozin (N=408)	Empagliflozin (N=369)	Crude HR (95% CI)	P-value	Adjusted HR (95% CI)	P-value
Primary endpoints	53 (13.0%)	47 (12.7%)	0.98 (0.68-1.45)	0.92	1.01 (0.68-1.50)	0.96
Secondary endpoints						
CV death	12 (2.9%)	10 (2.7%)	0.94 (0.41-2.19)	0.89	1.01 (0.43-2.35)	0.98
All cause death	18 (4.4%)	20 (5.4%)	1.24 (0.66-2.35)	0.50	1.27 (0.67-2.41)	0.46
HF hospitalization	44 (10.8%)	39 (10.6%)	0.98 (0.63-1.50)	0.91	1.01 (0.65-1.54)	0.99
CV hospitalization	72 (17.6%)	59 (16.0%)	0.89 (0.63-1.26)	0.51	0.90 (0.64-1.27)	0.54

CV, cardiovascular; HF, heart failure; HR, hazard ratio; CI, confidence interval

Primary outcome indicated a composite of CV death or HF hospitalization..Adjusted HR is adjusted for age, sex, diabetes and chronic kidney disease

eTable 10. Baseline characteristics of the study cohort after inverse probability of treatment weighting

	Dapagliflozin (n = 3003)	Empagliflozin (n = 2515)	P value	SMD
Age	68.60 ± 13.39	68.60 ± 13.46	0.998	0.045
Sex			0.988	0.001
Male	1830 (60.9%)	1529 (60.9%)		
Female	1176 (39.1%)	982 (39.1%)		
Hypertension	878 (29.2%)	733 (29.2%)	0.987	0.001
Diabetes	1281 (42.6%)	1070 (42.6%)	0.988	<0.001
Renal function				
baseline	1.12 ± 0.75	1.11 ± 0.71	0.954	0.014
baseline function	786 (26.2%)	637 (25.4%)	0.504	0.009
f/u	1.24 ± 0.98	1.23 ± 0.98	0.638	NA
f/u function	921 (30.6%)	707 (28.2%)	0.044	NA
delta	0.12 ± 0.94	0.11 ± 0.84	0.636	NA
NT-proBNT				
baseline	3251.9 ± 6689.3	3242.8 ± 6689.7	0.960	0.190
f/u	3398.6 ± 8061.2	3265.1 ± 7546.4	0.526	NA
delta	146.6 ± 141.9	22.25 ± 132.1	0.521	NA
LVEF				
baseline	47.69 ± 15.07	47.68 ± 15.12	0.993	0.052
baseline (≤40)	1019 (33.9%)	890 (35.4%)	0.240	0.008
f/u	48.88 ± 0.26	48.77 ± 0.29	0.770	NA
f/u (≤40)	886 (29.5%)	768 (30.6%)	0.375	NA
delta	1.20 ± 0.16	1.09 ± 0.17	0.641	NA
BW				
baseline	67.92 ± 0.27	67.92 ± 0.30	0.998	0.051
f/u	65.67 ± 0.27	65.72 ± 0.30	0.894	NA
delta	(-2.25 ± 6.60)	(-2.20 ± 6.76)	0.769	NA
Medications				
BB	2496 (83.0%)	2039 (81.2%)	0.076	0.003
ARNI/ACEI/ARB	2594 (86.3%)	2154 (85.7%)	0.563	0.011
ARNI	1060 (35.3%)	884 (35.2%)	0.956	0.012
ACEI	151 (5.0%)	170 (6.8%)	0.006	0.011
ARB	1992 (66.3%)	1569 (62.5%)	0.003	0.011
MRA	1583 (52.7%)	1371 (54.6%)	0.162	0.010
Digoxin	365 (12.1%)	256 (10.2%)	0.022	0.003
Vasodilators	976 (32.5%)	743 (29.6%)	0.021	0.007
Ivabradine	225 (7.5%)	172 (6.9%)	0.369	0.007

© 2025 Bu S et al. *JAMA Network Open*

[a] impaired renal function means creatinine level > 1.2 mg/dL.

BB,beta blocker; BW, body weight; ARNI, angiotensin receptor/neprilysin inhibitor; ACEI, angiotensin converting enzyme inhibitor; ARB, angiotensin II receptor blocker; MRA, mineralocorticoid receptor antagonist; NA,not applicalble; SMD,standardized mean differences

eTable 11. Primary and secondary outcomes of IPTW patients

	Dapagliflozin (n = 3003)	Empagliflozin (n = 2515)	HR (95% CI)	P-value
Primary outcome	315 (10.5%)	237 (9.5%)	0.93 (0.79-1.10)	0.420
Secondary outcomes				
CV death	92 (3.1%)	67 (2.7%)	0.90 (0.66-1.23)	0.509
All cause death	170 (5.7%)	133 (5.3%)	0.97 (0.78-1.22)	0.809
HF hospitalization	255 (8.5%)	198 (7.9%)	0.96 (0.80-1.16)	0.678
CV hospitalization	418 (13.9%)	318 (12.7%)	0.94 (0.81-1.09)	0.390

CV, cardiovascular; HF, heart failure; HR, hazard ratio; CI, confidence interval

Primary outcome indicated a composite of CV death or HF hospitalization..Adjusted HR is adjusted for age, sex, diabetes and chronic kidney disease

© 2025 Bu S et al. *JAMA Network Open*

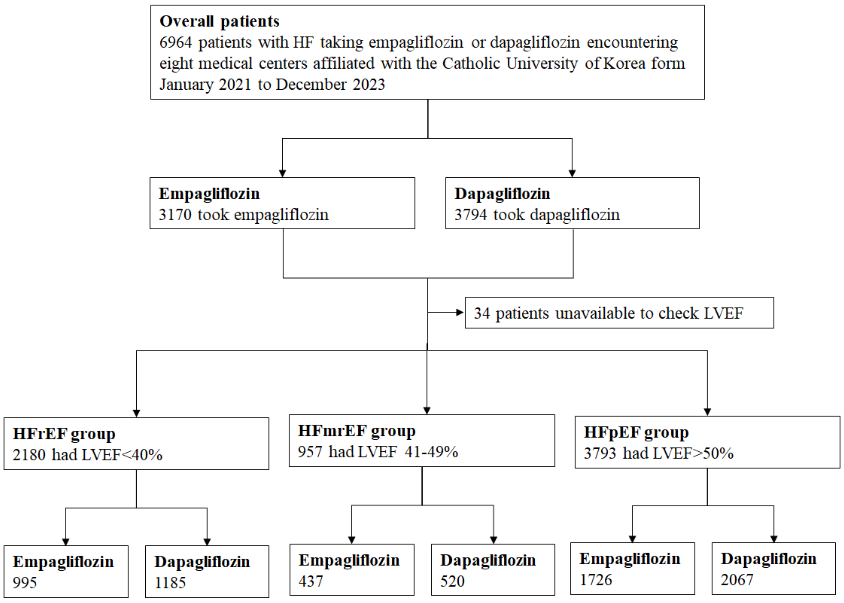

eFigure 1. Study flow chart

LVEF, left ventricular ejection fraction; HF, heart failure

© 2025 Bu S et al. *JAMA Network Open*

	Dapagliflozin	Empagliflozin	AHR (95% CI)	P-value	P for interaction
**Age**					0.251
<75	94/1510 (6.2%)	96/1491 (6.4%)	1.09 (0.82-1.45)	0.538	
≥75	147/955 (15.4%)	133/974 (13.7%)	0.92 (0.73-1.17)	0.509	
**Sex**					0.924
Male	132/1476 (8.9%)	125/1468 (8.5%)	1.00 (0.78-1.28)	0.987	
Female	109/989 (11.0%)	104/997 (10.4%)	0.99 (0.76-1.29)	0.932	
**HTN**					0.062
Yes	89/737 (12.1%)	68/739 (9.2%)	0.80 (0.58-1.10)	0.170	
No	152/1728 (8.8%)	161/1726 (9.3%)	1.12 (0.90-1.40)	0.312	
**DM**					0.228
Yes	90/1002 (9.0%)	73/1003 (7.3%)	0.83 (0.61-1.14)	0.248	
No	151/1463 (10.3%)	156/1462 (10.7%)	1.09 (0.87-1.36)	0.454	
**CKD**					0.295
Yes	98/619 (15.8%)	103/610 (16.9%)	1.13 (0.86-1.49)	0.386	
No	143/1846 (7.7%)	126/1855 (6.8%)	0.90 (0.71-1.14)	0.385	
**LVEF**					0.365
HFrEF	126/844 (14.9%)	132/855 (15.4%)	1.06 (0.83-1.35)	0.640	
HFmrEF	17/343 (5.0%)	22/350 (6.3%)	1.30 (0.69-2.45)	0.414	
HFpEF	98/1278 (7.7%)	75/1260 (6.0%)	0.82 (0.60-1.10)	0.182	
**DM CKD**					0.759
Yes	31/237 (13.1%)	34/255 (13.3%)	1.08 (0.66-1.75)	0.768	
No	210/2228 (9.4%)	195/2210 (8.8%)	0.98 (0.81-1.19)	0.848	
**DM HFrEF**					0.592
Yes	33/235 (14.0%)	32/213 (15.0%)	1.02 (0.62-1.66)	0.941	
No	208/2230 (9.3%)	197/2252 (8.7%)	0.99 (0.81-1.20)	0.875	
**CKD HFrEF**					0.136
Yes	56/249 (22.5%)	63/245 (25.7%)	1.24 (0.86-1.78)	0.247	
No	185/2216 (8.3%)	166/2220 (7.5%)	0.93 (0.75-1.15)	0.493	

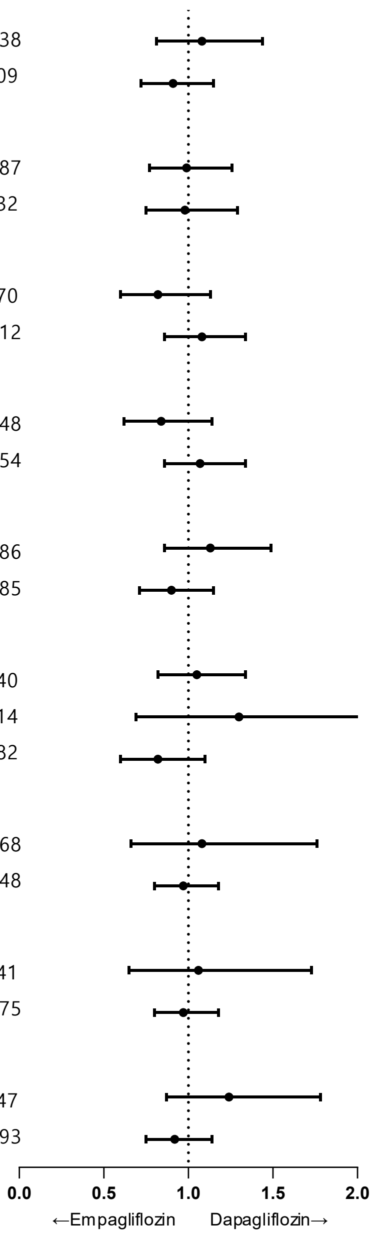

eFigure 2. Comparison of the effect of dapagliflozin and empagliflozin on the primary outcome in the subgroups of the propensity score-matched cohort.

CKD means baseline Cr > 1.2 mg/dL, HFrEF means baseline LVEF ≤ 40%..

CKD, chronic kidney disease; Cr, creatinine; DM, diabetes mellitus; HFrEF, heart failure with reduced ejection fraction

© 2025 Bu S et al. *JAMA Network Open*

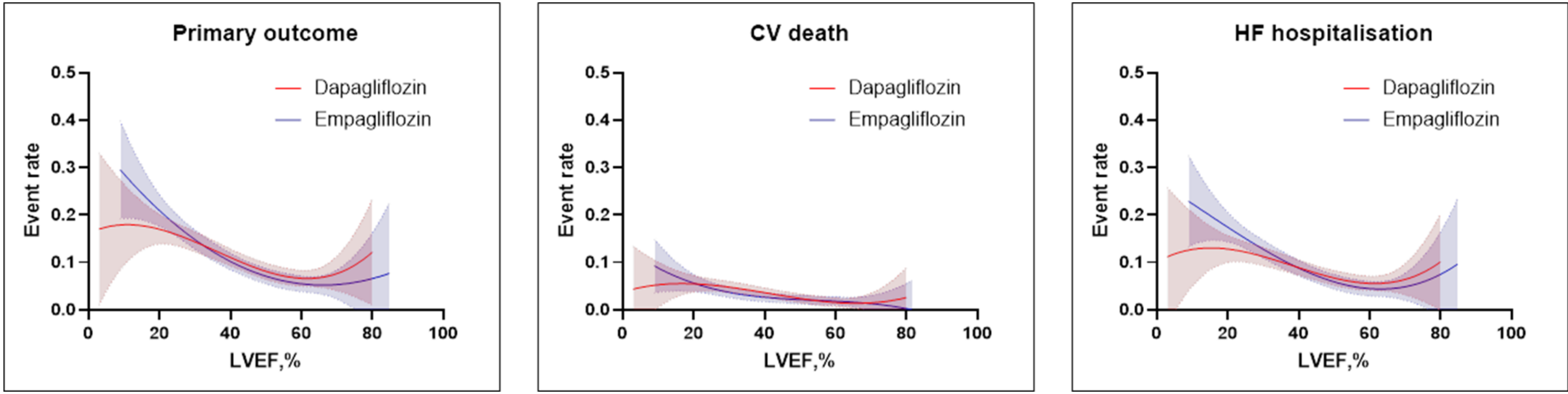

eFigure 3. Spline curve of event rates

LVEF, left ventricular ejection fraction; CV, cardiovascular; HF, heart failure

© 2025 Bu S et al. *JAMA Network Open*

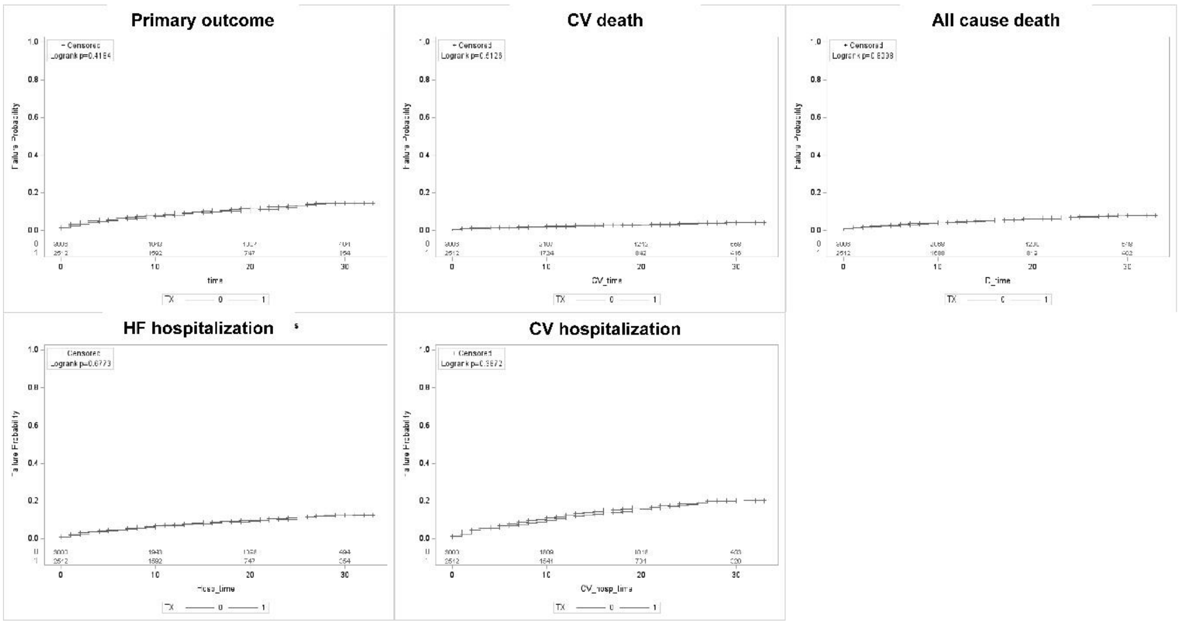

eFigure 4. Primary and secondary outcomes of KM curve using IPTW

LVEF, left ventricular ejection fraction; CV, cardiovascular; HF, heart failu

© 2025 Bu S et al. *JAMA Network Open*
