# Supplementary material for: Empagliflozin and Dapagliflozin Outcomes in Heart Failure
Source: JAMA Netw Open. 2025 Dec 4;8(12):e2546865. doi: 10.1001/jamanetworkopen.2025.46865 (PMC12679325; doi:10.1001/jamanetworkopen.2025.46865)
Supplement: Supplement 2. — Data Sharing Statement [file jamanetwopen-e2546865-s002.pdf]

Data Sharing Statement

Bu. Empagliflozin and Dapagliflozin Outcomes in Heart Failure. *JAMA Netw Open*. Published December 04, 2025. doi:10.1001/jamanetworkopen.2025.46865

Data
Data available: No

Additional Information
Explanation for why data not available: We planned to continue followup and substudy. Until the end, no individual participant data will be available.
